# Need for nursing care after laparoscopic and open colorectal cancer surgery: a claims data analysis in German primary care

**DOI:** 10.1007/s00423-022-02592-8

**Published:** 2022-06-27

**Authors:** Jonas D. Senft, Benedikt B. Brück, Regina Poß-Doering, Thomas Bruckner, Joachim Szecsenyi, Beat P. Müller-Stich, Gunter Laux

**Affiliations:** 1 Department of General Practice and Health Services Research, University Hospital Heidelberg, Im Neuenheimer Feld 130.3, 69120 Heidelberg, Germany; 2 Institute of Medical Biometry and Informatics, University of Heidelberg, Heidelberg, Germany; 3 Department of General, Visceral and Transplant Surgery, University of Heidelberg, Im Neuenheimer Feld 420, 69120 Heidelberg, Germany

**Keywords:** Nursing care, Claims data analysis, Colorectal cancer, Minimally invasive surgery, Laparoscopic surgery

## Abstract

**Purpose:**

Our study analyzes the influence of minimally invasive vs. open surgery on the postoperative need for nursing care in patients with colorectal carcinoma. Colorectal cancer is an age-related disease, and oncologic surgery is increasingly performed in elderly patients. Long-term effects of the procedural choice on patients’ self-sufficiency and autonomy have not been scientifically addressed so far.

**Methods:**

Multivariable logistic regression models based on claims data from a statutory health insurer (AOK, Baden-Württemberg, Germany) were applied to assess potential risk factors for assignment patients to a nursing care level, a German scale to categorize individual need for nursing care, at 12 and 36 months after colorectal cancer surgery.

**Results:**

A total of 3996 patients were eligible to be included in the analysis. At 36 months postoperatively, 44 of 427 (10.3%) patients after minimally invasive colon cancer surgery and 231 of 1287 (17.9%) patients after open procedure were newly graded into a nursing care level (OR = 0.62, 95%CI = 0.44–0.90, *p* = 0.010). Thirty-four of 251 (13.5%) patients receiving minimally invasive rectal cancer surgery compared to 142 of 602 (23.6%) patients after open approach were newly assigned to a nursing care level (OR = 0.53, 95%CI = 0.34–0.81, *p* = 0.003).

**Conclusions:**

Laparoscopically assisted resection of colorectal cancer seems to be superior in preserving physical autonomy of elderly patients with colorectal cancer.

## Introduction

Colorectal cancer is an age-related disease which increasingly requires oncologic surgery in elderly patients [1, 2]. More than 70% of patients diagnosed with colorectal cancer are aged 65 years or older [3]. Consequently, oncological treatment has to be tailored for elderly patients. In particular, treatment strategies should seek to preserve self-sufficiency and functional autonomy, which are cornerstones for quality of life. However, knowledge about the impact of oncological colon cancer surgery on patients’ functional autonomy is scarce and has not been scientifically addressed so far.

Due to well-proven short-term benefits like reduction of postoperative pain and faster recovery, guidelines recommend laparoscopically assisted resection of colorectal cancer as an alternative for open surgical procedures [4–7]. Nevertheless, in clinical routine, open surgery still remains a mainstay in the treatment of colorectal cancer. Downsides of open procedures, such as more pain or prolonged recovery, are often accepted as trade-off for shorter operation time and assumed better oncological outcome, although according to previous RCTs, the oncologic outcome of laparoscopically-assisted surgery seems non-inferior to open surgery [8–14]. Furthermore, the cost-effectiveness of laparoscopic oncological procedures is still being questioned [15].

So far, long-term effects of the procedural choice on patients’ self-sufficiency and functional autonomy have not been scientifically addressed. Consequently, and to our knowledge as a first, this claims data analysis aimed to answer the question whether the choice of surgical approach may have an effect on post-operative nursing care needs in patients with colorectal cancer.

## Methods

### Study design

A cohort study was conducted. Claims data related to patients treated in German primary care recorded between January 1, 2011 and December 31, and 2017 were supplied by the AOK statutory health insurance company (AOK Baden-Wuerttemberg, Germany). Ethical approval for this analysis was given by the local institutional Ethics Committee of the University Hospital Heidelberg (No. S-460/2020).

### Study population and data acquisition

Data were recorded by the AOK statutory health insurance company for reimbursement purposes and continuous evaluation of the HZV program, a comprehensive continuous evaluation program of general practitioner-centered care in German primary care (German: “Hausarztzentrierte Versorgung” (HZV)) [16]. For the analysis, data were supplied by the AOK to the Department of General Practice and Health Services Research at Heidelberg University Hospital. Subjects could not be identified, neither directly or through identifiers linked to the subjects. Data storage and extraction were performed with MySQL Community Server × 64 (Oracle Corporation, Redwood Shores, CA, USA). All national and institutional guidelines concerning data acquisition for retrospective analyses were followed at all times. The obtained dataset comprised age, gender, diagnoses according to ICD-10 coding as well as accounting data on consultations, prescribed medication, and hospital stays. Patient data were included into the analysis if they were aged 65 years or older and underwent non-emergency surgery for non-metastatic colorectal cancer. Cases were identified from the supplied data using the ICD-10 diagnosis (C18, C19, C20) and the German version of the International Classification of Procedures in Medicine (OPS, German: Operationen- und Prozedurenschlüssel) [17].

### Outcome parameters

To evaluate the effect of surgery on long-term need for nursing care, the nursing care level was assessed for each patient at 12 and 36 months after surgery. In Germany, the nursing care level is determined via a classification provided by the Medical Control Service (German: “Medizinischer Kontrolldienst”), an independent and governmentally supervised institution for assessment in health services. The evaluation for the nursing care level is usually initiated by patients, relatives, or family physicians, if affected patients need nursing care support. Upon assessment by experts from the Medical Control Service, the current individual need for nursing care and household assistance is categorized on a scale of 1 to 3 reflecting the individual care dependency: minor (I), average daily need of care at least 45 min; moderate (II), average daily need of care at least 120 min; and severe (III), average daily need of care at least 240 min and round-the-clock support, e.g., permanent bed confinement. The classification is based on the degree of personal limitation in daily life. Mobility (e.g., mobility within the domestic environment), cognitive ability (e.g., orientation), communication, psychological problems (e.g., agitation at night), self-care (e.g., independent personal hygiene), dealing with illness-related requirements (e.g., taking medication independently), and the organization of everyday life and social contacts (e.g., organizing daily routines) are assessed.

The following associated factors were determined for the analysis in the multivariable regression model: surgical procedure, age, gender, and morbidity according to Charlson index, a sum score determined according to ICD-10 diagnoses assigned to values between 1 and 6 according to severity to approximate patients’ overall morbidity [18]. Surgical procedure, lymph node metastasis, and metachronous metastasis were identified via ICD-10 coding. Application of neoadjuvant or adjuvant chemotherapy was determined by records of the central pharmaceutical numbers of prescribed medications (“Pharmazentralnummer”, PZN).

### Statistical analysis

Statistical analysis was performed using SPSS for Windows (IBM 27 Armonk, NY: IBM Corp). Chi-square test for categorical data and *T*-test for continuous data were used to assess differences between patients undergoing open or laparoscopically assisted surgical approach. Assignment to a nursing care level was analyzed 12 and 36 months after surgery. Influence of examined factors on these two dependent binary outcomes was determined by multivariable binary logistic regression. To assess the independence among variables, correlation tests were performed using the Phi coefficient for the dichotomic factors, the chi square test for ordinal variables, and the Eta coefficient for continuous parameters. For all analyses, results were considered statistically significant, if the *p* value was 0.05 or less.

## Results

**Fig. 1:**
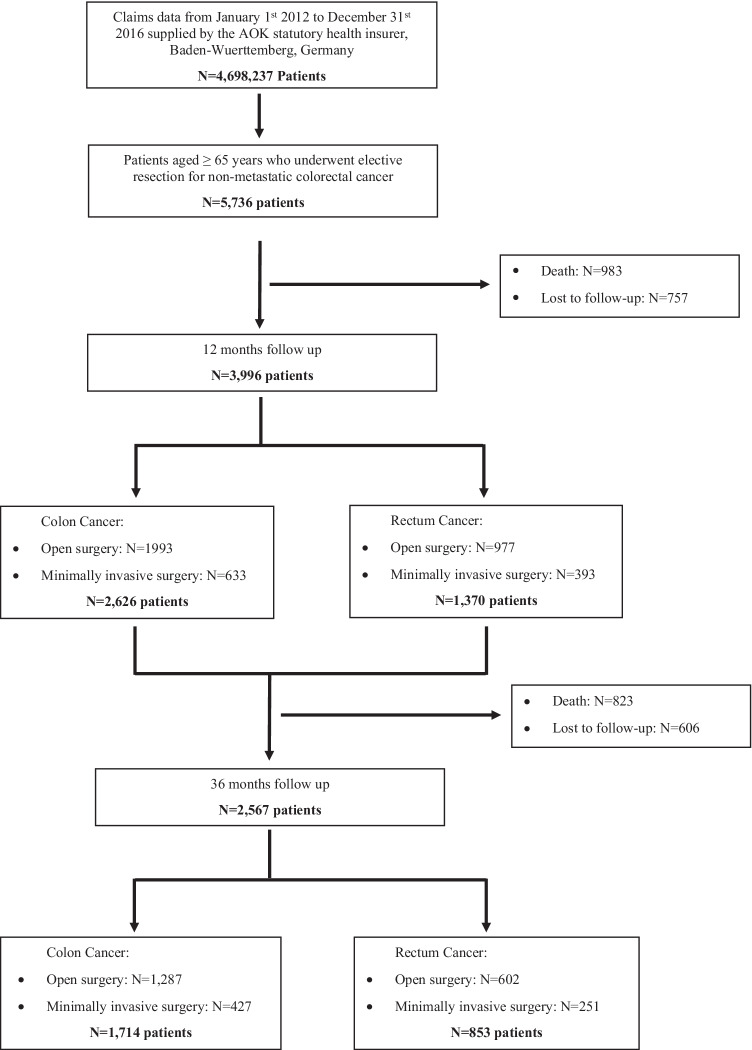
The flowchart shows the selection and sorting of claims data cases according to the inclusion and exclusion criteria

A total of 3996 patients were eligible to be included in the analysis. Figure 1 describes the study population in a flowchart. The baseline characteristics of the included patients are shown in Table 1.

**Table 1:** Baseline characteristics

		Colon cancer					Rectal cancer	
		*Open*	*Minimal invasive*			*Open*	*Minimal invasive*	
		(*N* (%))	(*N* (%))	*p* value		(*N* (%))	(*N* (%))	*p* value
Patients (*N*)		1,993 (75.9)	633 (24.1)			977 (71.3)	393 (28.7)	
Age (years)		77.6 ± 6.6	76.4 ± 6.2	< 0.001		75.8 ± 6.2	75.6 ± 6.5	0.557
Sex (m)		986 (49.5)	330(52.1)	0.244		603 (61.7)	225 (57.3)	0.126
Charlson Index*		5.6 ± 2.7	5.3 ± 2.7	0.351		5.9 ± 2.7	5.4 ± 2.6	0.267
Nursing care level†								
	None	1,842 (92.4)	598 (94.5)	0.167		922 (94.4)	377 (95.9)	0.364
	I	113 (5.7)	23 (3.6)			41(4.2)	14 (3.6)	
	II	35 (1.8)	12 (1.9)			12 (1.2)	1 (0.3)	
	III	3 (0.2)	0 (0)			2 (0.2)	1 (0.3)	
Surgical procedure								
	Ileocecal resection	24 (1.2)	9 (1.4)	0.669	Anterior resection	298 (30.5)	139 (35.4)	0.080
	Right hemicolectomy	1,252 (62.9)	335 (52.9)	< 0.001	Deep anterior resection	498 (51.0)	180 (45.8)	0.083
	Transverse colectomy	64 (3.2)	8 (1.3)	0.009	Abdominoperineal resection	178 (18.2)	73 (18.6)	0.878
	Left hemicolectomy	286 (14.4)	116 (18.3)	0.016	Proctocolectomy	3 (0.3)	1 (0.3)	0.870
	Sigmoid resection	254 (12.8)	142 (22.4)	< 0.001				
	Total colectomy	100 (5.0)	10 (1.6)	< 0.001				
	Segmental resection	13 (0.7)	13 (2.1)	0.002				
Stoma placement								
	Ileostomy	15 (0.8)	2 (0.3)	0.233		2 (0.2)	1 (0.3)	0.859
	Colostomy	78 (3.9)	7 (1.1)	0.001		108 (11.0)	24 (6.1)	0.005
Lymph node metastasis		96 (4.8)	42 (6.6)	0.074		62 (6.3)	23 (5.9)	0.732
Chemotherapy		516 (25.9)	146 (23.1)	0.161		362 (37.1)	135 (34.3)	0.347

Continuous values are presented as mean and standard deviation; *m* male

^*^The Charlson index is a sum score determined according to ICD-10 diagnoses assigned to values between 1 and 6 according to severity to approximate patients’ overall morbidity[18]

^†^Nursing care level is a classification determined by independent experts to clarify care insurance claims for individuals in need of care in Germany. Based on special criteria, those in need are assigned to one of three grades. Grade 3 corresponds to the highest need for care

Independence of included factors in the regression models was examined by correlation tests. There was low correlation between the influencing factor “minimal invasive surgery” and influencing factors “age” and “colostomy.” However, both factors do not exceed a correlation value of 0.1. Therefore, the included factors can be considered independent among each other.

### Colon cancer

At 12 months postoperatively, 283 of 1993 (14.2%) patients who underwent open surgery and 35 of 633 (5.5%) patients after minimally invasive surgery were newly categorized into a nursing care level. The corresponding odds ratio was significantly lower for patients who underwent minimal invasive surgery compared to open surgery (OR = 0.42, 95%CI = 0.29–0.61, *p* < 0.001). Rising age (OR = 1.10, 95%CI = 1.08–1.13, *p* < 0.001), male gender (OR = 1.33, 95%CI = 1.03–1.72, *p* = 0.030), an increased morbidity index (OR = 1.15, 95% CI = 1.10–1.20, *p* < 0.001), and the placement of ileostomy (OR = 8.36, 95%CI = 2.87–24.33, *p* < 0.001) or enterostomy (OR = 3.46, 95%CI = 2.11–5.69, *p* < 0.001) were associated with a higher risk of being categorized into a nursing care level.

At 36 months postoperatively, 231 of 1287 (17.9%) patients after open surgery and 44 of 427 (10.3%) patients after minimally invasive surgery were newly categorized into a nursing care level. The corresponding odds ratio remains significantly lower for patients who underwent minimal invasive surgery compared to open procedure for colon cancer (OR = 0.62, 95%CI = 0.44–0.90, *p* = 0.010). Associated risk factors for being assigned to a nursing care level at 36 months were rising age (OR = 1.10, 95%CI = 1.07–1.12, *p* < 0.001), male gender (OR = 1.58, 95%CI = 1.18–2.11, *p* = 0.002), increased morbidity index (OR = 1.20, 95%CI = 1.14–1.26, *p* = 0.004), and placement of an ileostomy (12.36, 95%CI = 2.26–67.52, *p* = 0.004) or enterostomy (3.22, 95%CI = 1.65–6.30, *p* = 0.001) (Table 2).

**Table 2:** Colon cancer: results of the multivariable analysis of patients graded into a nursing care level within 1 year of follow-up

		*12 months follow-up* *N* = 2626		*36 months follow-up* *N* = 1714			
	*OR*	*(95% CI)*	*p*		*OR*	*95% CI*	*p*
Minimal invasive surgery	0.418	(0.288, 0.609)	< 0.001		0.624	(0.435, 0.896)	0.010
Age	1.103	(1.080, 1.127)	< 0.001		1.097	(1.071, 1.124)	< 0.001
Gender (female)	1.331	(1.028, 1.723)	0.030		1.579	(1.182, 2.110)	0.002
Charlson Index*	1.149	(1.097, 1.203)	< 0.001		1.198	(1.138, 1.261)	< 0.001
Ileostomy	8.357	(2.871, 24.328)	< 0.001		12.356	(2.261, 67.522)	0.004
Colostomy	3.459	(2.105, 5.686)	< 0.001		3.219	(1.645, 6.296)	0.001
Lymph node metastasis	0.933	(0.533, 1.635)	0.810		0.883	(0.335, 2.332)	0.802
Chemotherapy	0.815	(0.573, 1.161)	0.258		0.723	(0.488, 1.071)	0.723
Metachronous distant metastasis	1.370	(0.931, 2.017)	0.110		1.427	(0.934, 2.181)	0.101

Continuous values are presented as mean and standard deviation

*OR* odds ratio, *CI* confidence interval

^*^The Charlson index is a sum score determined according to ICD-10 diagnoses assigned to values between 1 and 6 according to severity to approximate patients’ overall morbidity[18]

### Rectal cancer

At 12 months after surgery for rectal cancer, 198 of 977 (20.3%) patients were newly categorized into a nursing care level after open surgery compared to 54 of 393 (13.7%) patients after minimally invasive approach. The corresponding odds ratio was significantly lower for patients who underwent minimal invasive surgery compared to open surgery (OR = 0.68, 95%CI = 0.48–0.96, *p* = 0.030), Rising age (OR = 1.09, 95%CI = 1,06–1.12, *p* < 0.001), an increased morbidity index (OR = 1.16, 95%CI = 1.10–1.22, *p* < 0.001), the placement of colostomy (OR = 2.21, 95%CI = 1.47–3.34, *p* < 0.001), and adjuvant chemotherapy (OR = 0.62, 95%CI = 0.44–0.87, *p* = 0.006) were associated risk factors for being assigned to a nursing care level.

At 36 months postoperatively, 142 of 602 (23.6%) patients were newly categorized into a nursing care level after open surgery compared to 34 of 251 (13.5%) patients after minimally invasive approach. The corresponding odds ratio of being categorized into a nursing care level remained lower for minimal invasive surgery compared to open surgery (OR = 0.53, 95%CI = 0.34–0.81, *p* = 0.003). Associated risk factors for patients being assigned to a nursing care level at 36 months were rising age (OR = 1.08, 95%CI = 1.05–1.15, *p* < 0.001), *p* = 0.002), increased morbidity index (OR = 1.15, 95%CI = 1.07–1.23, *p* < 0.001), placement of colostomy (2.83, 95%CI = 1.63–4.92, *p* < 0.001), and occurrence of metachronous distant metastasis (1.86, 95%CI = 1.15–3.01, *p* < 0.001) (Table 3).

**Table 3:** Rectal cancer: results of the multivariable analysis of patients graded into a nursing care level within 1 year of follow-up

		*12 months follow-up* *N* = 1370		*36 months follow-up* *N* = 853			
	*OR*	*(95% CI)*	*p*		*OR*	*95% CI*	*p*
Minimal invasive surgery	0.680	(0.481, 0.962)	0.030		0.527	(0.343, 0.809)	0.003
Age	1.087	(1.061, 1.115)	< 0.001		1.081	(1.047, 1.115)	< 0.001
Gender (female)	1.039	(0.766, 1.408)	0.807		1.095	(0.756, 1.586)	0.631
Charlson Index*	1.159	(1.097, 1.224)	< 0.001		1.150	(1.074, 1.231)	< 0.001
Ileostomy	4.199	(0.373, 47.259)	0.245		n/a	n/a	n/a
Colostomy	2.214	(1.470, 3.336)	< 0.001		2.830	(1.629, 4.918)	< 0.001
Lymph node metastasis	0.781	(0.414, 1.475)	0.447		0.781	(0.351, 2.198)	0.781
Chemotherapy	0.620	(0.440, 0.874)	0.006		0.841	(0.557, 1.271)	0.412
Metachronous distant metastasis	1.111	(0.728, 1.697)	0.626		1.861	(1.149, 3.013)	0.012

Continuous values are presented as mean and standard deviation

*OR* odds ratio, *CI* confidence interval

^*^The Charlson index is a sum score determined according to ICD-10 diagnoses assigned to values between 1 and 6 according to severity to approximate patients’ overall morbidity [18]

## Discussion

For the first time, the long-term effect of laparoscopic versus open surgery for colorectal cancer on postoperative nursing care needs was evaluated in this present study. The multivariable analysis showed that the minimally invasive approach for colon as well as for rectum cancer is associated with a lower risk of needing nursing care after 12 and 36 months of follow-up.

Since colorectal cancer is a disease with an age-dependent incidence, the results of this analysis are of high relevance for its oncological treatment pathways for elderly patients. In clinical routine, colorectal surgery is still frequently performed by open procedure, particularly in technically challenging resections of locally advanced tumors or operable distant metastasis, although it has been shown that laparoscopically assisted surgery may be performed safely and with comparable oncological outcome [19–22]. According to the results of this analysis, preferring minimally invasive surgery for colorectal cancer in elderly patients may contribute to preserve physical autonomy. While short-term benefits of laparoscopically assisted colorectal surgery, e.g., early mobilization, reduced postoperative pain or early return of bowel function are well-known [4–7], the results of this study indicate that advantages of the minimally invasive approach exceed effects on short-term morbidity in elderly patients and may have lasting healthcare-related effects, which have not been identified so far. The reason for this finding may be hypothesized to lie within the reduced functional reserve of elderly patients to respond to surgical trauma. Frailty is state of multifactorial decline in physiologic function with a prevalence of 25–50% for patients over 80 years and is associated with high vulnerability to sudden severe health status changes [23–26]. In surgery, frailty has been shown to be associated with higher postoperative morbidity and mortality, e.g., after vascular surgery, kidney transplantation, and general surgery or emergency laparotomy [27–30]. Particularly in frail patients, the extent of abdominal incisional trauma may be a decisive factor affecting long-term disability. Severe postoperative pain, slow recovery of bowel function, wound healing disorders, immobilization, and prolonged hospital stay may lead to further morbidity and weakening of general condition, which could tip the scale for patients on the verge of self-sufficiency. These potential effects of open oncologic surgery should be taken into account when choosing an optimal surgical treatment. Postoperative loss of autonomy should be considered as a highly relevant outcome after surgery in elderly patients, particularly with regard to its potential impact on quality of life. The effects of the invasiveness on a postoperative need for nursing care should be clearly explained to patients and incorporated in preoperative counseling and choice of the surgical procedure.

Besides the invasiveness of surgery and unalterable risk factors like patients’ age, morbidity, or gender, the multivariable analysis showed that placement ileostomy or colostomy has a marked effect on the postoperative nursing care needs in colorectal cancer surgery. It is not surprising that stoma management particularly in older patients may require nursing care assistance and therefore contributes to loss of self-sufficiency in many cases, particularly in ileostomy which is known for difficulties in postoperative management due to fluid loss. On the other hand, it has to be mentioned that the descriptive analysis shows a higher rate of colostomy for open surgery of colon as well as rectum. Since stoma is placed particularly in the setting of emergency or complicated surgery which may be associated with a worse outcome, confounding may also be considered in this regard. While cases admitted to hospital as emergency were excluded from the analysis, subacute emergency cases or complicated surgery are not captured by claims data. However, with regard to ostomy and advance of oncologic disease, the results of our analysis suggest that stoma placement in elderly patients should be applied after particularly careful evaluation, and stoma management should best be addressed by comprehensive preoperative instructions to patient and nursing care providers to mitigate postoperative challenges.

To our knowledge, this is the first study evaluating the long-term effects of laparoscopic versus open surgery for colorectal cancer on postoperative need for nursing care. Limitations of this study lie within the nature of claims data. We deliberately chose claims data for this analysis due to high statistical power and its possibilities to facilitate multivariable models. Selection bias regarding the choice of surgical procedure cannot be excluded completely. However, both groups are homogeneous in terms of morbidity. The nursing care level and age differs only modestly for patients with colon cancer. The supplied claims data did not include information regarding tumor stage and frailty. However, all available variables referring to the stage of colorectal cancer have been included in the multivariable analysis by assessing lymph node involvement, occurrence of metastases, and application of adjuvant chemotherapy. Since multimorbidity and age are strong predictors for frailty [25], these factors were included in the regression model.

## Conclusion

As a conclusion, this analysis provides evidence that the risk of needing long-term nursing care after colon and rectal cancer surgery is associated with the invasiveness of procedural choice. Laparoscopically assisted resection of colorectal cancer seems superior to preserve the autonomy of patients and may be preferred over open surgery in elderly patients. Ileostomy is associated with a marked effect on the postoperative need for nursing care and should be avoided if possible. To preserve autonomy and quality of life in elderly patients, these procedural risk factors should be taken into account in preoperative counseling and choosing of the surgical approach. Future studies should routinely address the need for nursing care as a relevant outcome after surgery.

## Data Availability

The datasets generated and/or analyzed during the current study are available from the corresponding author on reasonable request.
